# Do Physical Activity and Diet Independently Account for Variation in Body Fat in Children and Adolescents? A Systematic Review Unpacking the Roles of Exercise and Diet in Childhood Obesity

**DOI:** 10.3390/nu17233779

**Published:** 2025-12-02

**Authors:** Richard D. Telford, Sisitha Jayasinghe, Nuala M. Byrne, Rohan M. Telford, Andrew P. Hills

**Affiliations:** 1 Research Institute for Sport and Exercise, University of Canberra, Bruce, ACT 2617, Australia; 2 Health Research Institute, University of Canberra, Bruce, ACT 2617, Australia; 3 Independent Researcher, Newcastle NE1 1EN, UK; sisitha.jay@gmail.com; 4 School of Health Sciences, UTAS Health, University of Tasmania, Launceston, TAS 7250, Australia; nuala.byrne@utas.edu.au (N.M.B.); andrew.hills@utas.edu.au (A.P.H.); 5 National Centre for Epidemiology and Population Health, Australian National University, Canberra, ACT 2601, Australia; rohan.telford@anu.edu.au

**Keywords:** childhood obesity, adolescents, diet, physical activity, body composition, percent body fat, BMI, energy intake, dietary surveys, energy expenditure, sedentarism

## Abstract

**Background/Objectives:** Physical activity (PA) and energy intake (EI) are central targets of community initiatives to reduce the prevalence of childhood obesity. The general effects of PA and EI in influencing energy balance and body composition are clear. However, the independent impacts of PA and EI on the adiposity of children growing up amidst westernized lifestyles are inconclusive, as few studies have employed sufficiently robust methodology to provide solid independent associative data. **Methods:** We carried out a systematic review of the research addressing the independent associations of adiposity with each of PA and EI in free-living town or city-dwelling children and adolescents. Acceptable publications included objective measures of fat mass and PA, best standard practice EI assessments, and appropriate statistical modeling. **Results:** Of approximately 700 publications explored, only four satisfied all the pre-set methodological standards. All four studies involved predominantly White participants from westernized cities and had the same outcomes. Adiposity was strongly independently and negatively related to PA, but there was no evidence of any independent relationship between adiposity and EI. Potential misreporting was considered, especially under-reporting by participants with greater adiposity, butpost-hoc assessments were unable to find any evidence that this influenced the outcomes. **Conclusions:** In general, children with higher adiposity consumed no more food and beverage energy than their leaner counterparts, but they were less active. However, despite some support for the validity of the commonly used and validated EI assessments, their subjective nature raises the possibility that inaccuracy masked relationships. Additional well-designed research is needed, and notwithstanding the vital role that sound nutrition plays in the healthy development of our youth, the consistency of outcomes of the well-executed studies in this review suggests that campaigns targeting youth obesity would benefit from strategies focusing strongly on increasing PA.

## 1. Introduction

Childhood and adolescent overweight and obesity have emerged as pressing global concerns in contemporary times [1]; increased adiposity is generally the result of a chronic imbalance between dietary energy intake (EI) and energy expenditure. The complexity surrounding body composition control is now well established, with adiposity, energy consumption, and expenditure all influenced by biological, developmental, behavioral, genetic, and environmental factors [2]. However, a fundamental question arises as to the extent to which the modifiable lifestyle factors, EI and physical activity (PA), influence the development of adiposity. This question is yet to be resolved, but lifestyle management of body composition is of particular relevance, especially to clinicians and health workers who are tasked with designing government campaign strategies to reduce the prevalence of obesity across the population.

With childhood obesity being a major global concern [3], and evidence that youth who are overweight and/or obese are five times more likely to become obese adults [4], it is prudent to gain a better understanding of the influences of EI and PA on these trends. If current trajectories continue unchecked, conservative estimates suggest that by 2050, approximately 186 million children aged 5 to 14 worldwide could be living with obesity [5]. While targeted individual interventions are appropriate for cases of morbid obesity, broader public health efforts have historically taken a dual approach, focusing on both reducing EI as well as increasing PA. Yet, despite campaigns being initiated across many countries and jurisdictions, such as Australia’s “Life. Be in it”, initiated by the Victorian Government 50 years ago, these initiatives have failed to make any meaningful impact on community-wide obesity rates, including those in children and adolescents [6].

This raises important questions regarding strategy. Should campaigns to reduce the prevalence of community obesity in children focus on both EI and PA as contributors to energy balance in a similar fashion, or might better returns be gained from focusing more on one aspect? Whilst a variety of factors are likely to contribute to the apparent lack of impact of community campaigns on the prevalence of obesity in our youth, a better understanding of the independent relationships of two major components of community campaigns, PA and EI, may assist future campaign strategies. Here, we review the reported evidence of independent relationships between adiposity and each of total daily PA and EI. We did not review the relative intensity of the PA or the macro and micro components of EI.

The data utilized in this review were derived from research conducted with sound methodology, minimizing the chance of incorrect outcomes and biased inferences. Publications involving avoidably subjective methodology for any one of the variables of interest (PA, EI, and adiposity) and, therefore potentially misleading, were excluded. An example of particular concern is the frequent use of the weight status measure body mass index (BMI, body mass/height^2^) as a proxy for adiposity, especially when longitudinal or serial data are involved, because mean increases in BMI in children and adolescents may occur without change [7], or even decreases [8] in percent body fat. Another key concern is the absence of statistical adjustment for the confounding effects of the explanatory covariables PA and EI; an absence likely to produce spurious correlations with the response variable adiposity.

## 2. Materials and Methods

**Figure 1:**
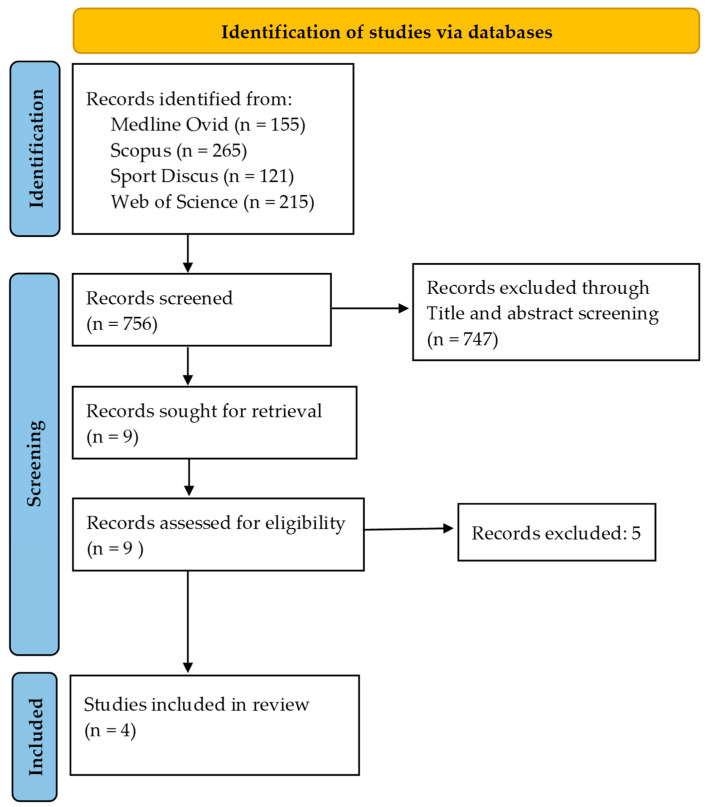
Study selection.

To examine the independent relationships between adiposity and both PA and EI, an extensive literature search was conducted for studies meeting predefined methodological criteria (outlined in Figure 1). Four major databases—Medline (Ovid), SPORTdiscus, Scopus, and Web of Science—were systematically searched, and the number of articles screened at each stage is presented in Figure 1. Ultimately, four studies met all methodological standards, as summarized in Table 1.

Only English-language studies were included in this review. Two independent investigators initially assessed each study’s eligibility based on predefined inclusion criteria. In instances of disagreement, a third researcher was consulted to reach a consensus. A comprehensive list of search concepts and an exemplary combination of search strings are available in Supplementary Table S1. As shown in Supplementary Table S1, risk of bias in the included studies was assessed using the ROBINS-E (Risk Of Bias In Non-randomized Studies—of Exposures) tool and evaluated for its confidence in risk of bias due to confounding variables, participant selection, intervention classification, missing data, outcome measurements, and reported outcomes. Insufficient or unreliable data within any required category disqualified studies from inclusion; as a result, only four of the approximately 700 initially identified articles satisfied all criteria. Our restriction of considered manuscripts to those written in English raises the possibility of overlooking some work satisfying our inclusion criteria.

A summary of pertinent aspects of these four studies (participants, interventions, measurements, analyses, and findings) was manually extracted and is shown in Table 2.

## 3. Results

### 3.1. Summary of Findings from Each Qualified Study (Listed Chronologically)

#### 3.1.1. The LOOK Pre-Adolescence Study 

This work [9] was conducted in 278 male and 256 female children, who completed all measures at 8 and 12 years of age. PA was assessed using pedometers, and percent body fat was determined using DEXA. EI at age 8 y was measured with a one-day dietary record based on previously described methods [10], where parents or guardians and teachers recorded all the foods, beverages, and supplements consumed by the children over a 24 h period on a school day, aided by detailed instructions, pictures, measuring cups, and spoons provided by the trained nutrition staff. At age 12 y, the method was extended to include two days of dietary records, a school day and a non-school day, based on the methodology adopted in the 2007 Australian National Children’s Nutrition and Physical Activity Survey, with parents and children interviewed by phone for approximately 30 min, and a software system-assisted data computation was used. Statistically, a general linear mixed methods model was used with adjustments for covariates PA and EI.

Cross-sectional relationships were essentially the same at age 8 and age 12 (where a one-day and two-day supervised dietary recall were utilized, respectively). Linear associations revealed that boys and girls with higher percent body fat were less physically active, both in terms of steps per day and moderate-and vigorous-intensity PA (*p* < 0.001 for both measures, both sexes). However, children with higher percent body fat did not consume more energy (nor fat, carbohydrate, or sugar). Boys with higher percent body fat consumed fewer carbohydrates (*p* = 0.01), with a strong trend towards consuming less energy (*p* = 0.05). Relationships were similar in both the lowest (leanest) and highest (fattest) quartiles, indicating that any influence of potential adiposity-related under-reporting was unlikely. Longitudinal analysis (combining data from both sexes) supported the cross-sectional findings, showing that children who reduced their PA over the four years increased their percent body fat (*p* = 0.04), with no evidence of any longitudinal relationships between percent body fat and EI.

#### 3.1.2. The Helena/European Youth Heart Study (EYHS) 

This study involved cross-sectional work with data from 1721 adolescent participants in the two projects, deriving independent relationships between adiposity and each of PA and EI [11]. PA was measured by accelerometry; EI by two 24 h recalls in the HELENA study and one 24 h recall in the EYHS; adiposity was measured by either skinfold thickness or BIA, DEXA, or ADP. Multilevel statistical analyses were used to examine the relationships among PA and EI and fat mass, with appropriate adjustments made for relevant covariates. The relationships between adiposity measurement, PA, and EI supported the cited authors’ hypothesis that more physically active and leaner adolescents have a greater EI than adolescents with relatively larger amounts of fat mass, but who were less physically active. Indeed, fat mass was negatively associated with energy intake in both studies and using four different measurement methods (*p* < 0.006)—body fat mass: skinfold thickness (*p* < 0.001), BIA (*p* < 0.001), Bod Pod (*p* ≤ 0.001), and DXA (*p* ≤ 0.002). The authors cited previous work in these cohorts, demonstrating that in adolescents from both the HELENA study and the EYHS, higher levels of physical activity were related to lower adiposity. Vigorous physical activity, but not moderate physical activity, was positively associated with energy intake in adolescents from the HELENA study, regardless of sex, age, pubertal status, or center (*p* < 0.001). In the EYHS, moderate physical activity, but not vigorous physical activity, was positively associated with energy intake, regardless of sex, age, pubertal status, fat mass assessed by skinfold thickness, or height (*p* ≤ 0.003). The association between PA and EI remained statistically significant even after participants with obesity (who are reportedly more likely to under-report EI) were excluded from the analyses.

#### 3.1.3. The Iowa Bone Development Study

This study focused on trajectories of obesity development in relation to trajectories of PA and EI [12]. There were 493 participants (51% females) who were assessed at least three times, from age 5 through to 19 years. PA was measured using accelerometry; adiposity by DEXA; and EI was assessed by validated dietary questionnaires (Block Kids’ questionnaire at Waves 3–6, and the National Cancer Institute Diet History Questionnaire II). The authors used a quartile rank within a wave as the EI indicator. Independent associations between the trajectory of adiposity development and the trajectories of PA and EI were investigated using a multivariable logistic regression model.

Participants with declining PA levels demonstrated significantly higher odds of becoming obese (adjusted odds ratio (OR) = 2.77 (95% CI = 1.16, 6.58). On the other hand, there was no relationship between EI and the odds of becoming obese. Indeed, the odds of increasing adiposity OR = 0. 74 (0.39, 1.40), and those for decreasing adiposity OR = 1.19 (0.61–2.30) were both trending in the opposite direction; increasing EI tending to be associated with decreased odds of becoming obese. This was clearer in children with a consistently high EI who had even lower odds of becoming obese (OR = 0.64 (0.22, 1.92), and children with a consistently low PA, who had higher odds of becoming obese (OR = 3.79 (1.31, 10.99).

#### 3.1.4. The LOOK Adolescence Study 

This study involved 556 (289 male) participants measured at age 12 years, 269 (123 males) of whom were measured again at age 16 years [8]. Their percent body fat was measured using DEXA; habitual PA, moderate- to vigorous-intensity PA by accelerometry; and EI was assessed with two (weekday and weekend) 24 h multi-pass recalls involving direct questioning and referencing to food photographs to prompt recall to refine the accuracy of the data collected. The likelihood of under-reporting of EI was minimized by adopting the Goldberg cut-off method [13] together with the post hoc finding that the relationships between adiposity, PA, and EI were unchanged in the range of lean children, who are less likely to under-report. General linear mixed modeling was used to generate independent relationships between the percent body fat and each of the PA and EI variables.

Percent body fat was significantly and independently negatively associated with PA. Cross-sectional (between-individual) analyses indicated that 10 min more of moderate- to vigorous-intensity PA per day was associated with a 0.6 lower %BF (95%CI 0.4–0.9, *p* < 0.001), independently of EI. In contrast, percent body fat was unrelated to total energy (*p* = 0.4), sugar intake (*p* = 0.2), or fat intake (*p* = 0.9). Longitudinal analyses were less well-powered but were, in general, supportive of the cross-sectional findings, with a trend toward a negative within-individual relationship between percent body fat and PA (*p* = 0.06). There was no evidence of any longitudinal relationships between percent body fat and EI.

## 4. Discussion

### 4.1. Review Outcomes

With only four studies satisfying the methodological standards considered to be sufficiently rigorous, the ability to draw definitive inferences is necessarily constrained. On the other hand, confidence is enhanced in that the outcomes of all four of the acceptable studies were remarkably consistent, despite variations in the methodology and population samples. In each sample of healthy young males and females, cross-sectional analyses showed adiposity to be strongly and negatively related to PA. However, no evidence of an independent relationship between adiposity and EI emerged in any of the studies. Longitudinal analyses were less decisive, but trends were consistent with the cross-sectional analyses; youth who reduced their PA levels over time tended to increase adiposity, and those who increased their PA tended to lower their adiposity. There remained no evidence of any longitudinal relationships between adiposity and EI.

### 4.2. Contrasts with Popular Opinion

The absence of evidence in support of a relationship between adiposity and EI does not match popular opinion. Indeed, the finding may appear counterintuitive, given that weight gain occurs through positive energy balance, which necessarily involves food and beverage consumption. However, it is important to consider the outcome of this review in the context of our specific question: “What is the strength of the independent relationship between adiposity and each of EI and PA in community-based children and adolescents?” The findings that PA, but not EI, explains the variation in youth adiposity across the community in each of the four studies of this review does not conflict with the fact that increasing EI without a change in energy output will increase adiposity.

Nevertheless, all other things being equal, the child or adolescent who consumes more energy than another in a given period of time is likely to store more body fat [14,15,16]. However, all things are not equal. The findings of the four qualified studies in this review suggest that in more physically active children and adolescents, the energy balance shifts to a less positive state (growing children generally are in positive energy balance), and so they have less fat storage. The findings also challenge a common supposition that children who are overweight or obese consume more food and beverages than their leaner counterparts; the evidence generated from this review suggests otherwise. The most physically active and leanest individuals had the highest EI from food and beverages.

### 4.3. Speculation from an Evolutionary Viewpoint

In seeking some rationale for the finding of a strong negative association between adiposity and PA, together with the failure to detect any association between adiposity and EI, we present some general speculation on evolutionary grounds. Human evolutionary-conserved neural circuitry tasked with balancing EI and energy output may have evolved to operate more effectively within an obligatory physically active environment. “Survival of the fittest” in this situation may have applied to individuals whose energy balance control favored a leaner body composition; thereby equipping them with superior agility and/or endurance, thus favoring survival from physical threats or food shortage. On the other hand, in periods of low physical threat, relative nutritional sufficiency, and minimal obligatory movement, neural control of the appetite might have evolved with a shift in energy balance towards energy storage, including increased adipose tissue. Consistent with this line of speculation linking appetite control with PA, muscular activity is known to influence appetite control, endocrine, and metabolism; for example, through increased insulin sensitivity [17], circulatory lactate increases, and modulated secretion of the appetite-regulating hormone acylated ghrelin [18].

### 4.4. Is the Null Relationship Correct or Are EI Measures Inadequate?

In looking to explain the lack of association between adiposity and EI in all four studies, we might suspect an adiposity-related misreporting bias. For example, under-reporting in individuals with overweight or obesity [13] will tend to offset the detection of any relationship between adiposity and EI. In the two LOOK studies and the Helena/EYHS work, the authors considered this potentially confounding issue. In the pre-adolescent LOOK study, the null association between adiposity and EI remained (as did the significant negative relationship between adiposity and PA) in the leanest quartile of the group, where under-reporting would not be expected. Similarly, in the Helena/EYHS study, removal of participants with obesity made no difference to the outcomes. The LOOK adolescent study provided further evidence that under-reporting was unlikely to be a concern, with application of the Goldberg method for offsetting the potential influence of under-reporting [13], which again had no effect on the finding of a null relationship between adiposity and EI.

One other way to check whether EI was sufficiently well measured to detect a relationship with adiposity is to consider whether EI was related to PA in any of the four studies. Indeed, such a relationship was evident in the HELEA study participants, where vigorous PA was positively related to EI (*p* < 0.05), and in the EYHS, where moderate PA was positively related to EI (*p* < 0.01). These relationships do support the premise that had there been a significant relationship between adiposity and EI in existence in these studies, it was likely to have been detected.

Nevertheless, despite general acceptance by dietary researchers [19], dietary records, recall, and food frequency questionnaires to assess EI are largely subjective and prone to inaccuracies. Even with multi-pass methodology involving systematic checking by direct communication with children and their parents and teachers by well-trained teams, and use of well-accepted software for computation of EI, these measures lack the objectivity and precision of a gold standard methodology, such as doubly labeled water [20]. However, the latter measure of total energy expenditure (from which EI is estimated) is costly, practically difficult to administer, and the conversion of energy expenditure to EI is also likely to introduce some error. In any case, inaccuracy of EI determination reduces the likelihood of detecting associations between EI and adiposity, and notwithstanding the support for the EI methodology outlined above, interpretation of the finding of a null relationship between EI and adiposity requires caution. Further investigations are required to substantiate or refute this finding, and this requires an objective assessment of EI.

### 4.5. Other Methodological Challenges

Importantly, we have underscored, without ambiguity, the methodological challenges faced by researchers who study childhood obesity. Subjectively acquired PA data, and the commonly used BMI as a proxy measure for adiposity, may satisfy research budgetary constraints, but insufficient reliability and validity of such data are likely to lead to misleading outcomes [7,9]. The other common, and potentially misleading, feature of many of the publications considered was an absence of statistical adjustment for PA or EI when determining the relationships of each of these covariates with adiposity. Consequently, spurious relationships are likely to be reported.

The selection of studies meeting the required methodological standards was relatively straightforward, but there were some well-conducted studies that were excluded because they did not specifically address the relationships between adiposity, PA, and EI. For example, the OPUS study [21] examined the independent cross-sectional and longitudinal associations between the fat mass index and PA with appropriate statistical modeling, but their analyses were confined to a measure of dietary energy density of food and beverage intake, rather than total EI. Of interest to the current review was its general consistency with the four included studies, with strong cross-sectional and longitudinal negative relationships reported between the fat mass index and PA after adjusting for energy density.

### 4.6. Community Campaigns

Our review findings can be considered in terms of community-based campaigns to reduce the general prevalence of childhood and adolescent obesity. Prior to our review, it had already been proposed that a more effective approach might be to place the major emphasis on PA, rather than on the restriction of EI [22]. Our review of well-designed research provides some support for this proposal. Indeed, the authors of the Helena/EYHS study [11] wrote that “although confirmation of our cross-sectional study results by prospective and experimental data is needed, our findings suggest that public health efforts aimed at promoting PA are more likely to be effective in preventing obesity than efforts designed to restrict EI”. For example, a national campaign, such as the above-mentioned Australian national “Life. Be in it” campaign, which focused equally on increasing PA and reducing EI, might benefit from focusing more on ways to increase PA and de-emphasizing the reduction of EI per se to focus on the consumption of healthy foods and beverages.

### 4.7. Limitations and Strengths

Inferences from this review must be considered within its limitations and strengths. Restricting studies to those adopting appropriate methodology and analyses is a strength, as we removed the studies that were most likely to have reported flawed outcomes. On the other hand, the paucity of eligible studies is a weakness. Even though this was counterbalanced to a degree by the unanimity of the outcomes, the eligibility of only four studies for inclusion thus limits confidence in drawing conclusions. As alluded to above, the subjectivity of EI assessment was a limiting factor. The authors recognized that this and provided evidence to suggest that under-reporting in overweight and obese children was unlikely to have had any effect on the outcome. One group’s investigation included measurements of the relationship between EI and PA, revealing their EI assessment to be at least of sufficient accuracy to reveal a statistically significant relationship. Nonetheless, the inherent subjectivity and potentially reduced signal-to-noise ratio in the EI determination constrain the interpretation of the inability to detect its relationship with adiposity. A final, clear limitation of our review was a lack of generalizability. The study’s participants were from towns and cities in affluent countries, and the participants were mostly White. Although subject homogeneity in each of the four studies might be regarded as a strength in terms of collective reporting, independent relationships of adiposity with EI and PA may vary in youth residing in nations of varying affluence, environments, ethnicities, nutrition, and PA opportunities. For example, for children living in an environment where a substantial level of daily PA is obligatory, but food is not in plentiful supply, EI may well emerge as a significant explanator of childhood adiposity.

### 4.8. Future Directions

That only four articles of approximately 700 considered possessed the required quality to measure independent relationships between childhood adiposity and each of EI and PA has obvious implications. More research employing high-quality methodology and appropriate statistical treatment is required. Absence of sufficient quality of any one of the four required measurements (adiposity, PA, EI, and statistical modeling) is likely to produce flawed outcomes. The weakest methodology of the four required characteristics is the largely subjective measurement of EI; therefore, future relatively large subject number population-based research would benefit from new approaches to reduce this subjectivity. With highly objective measures such as doubly labeled water being costly and impractical, new technologies involving remote sensing, image-based assessment, multisensory wearables, smartphone apps, and digital food records show promise, pending validation of EI assessment in children. Although not considered in the current review, future research might extend to investigating relationships of adiposity with macronutrient and/or micronutrient dietary composition, and with the quality of the PA, as well as sedentariness. Repeated measures and larger participant numbers are always an advantage, and along with more objective methodology, contribute to acquiring sufficient power to undertake longitudinal studies. Although still not permitting inferences of causation, in the absence of experimental research, longitudinal outcomes do provide a higher level of insight than those cross-sectional.

## 5. Conclusions

The scarcity of robust studies on the independent relationships of PA and EI with adiposity in youth underscores the need for further high-quality research. However, findings from four publications employing sound methodology were remarkably consistent, demonstrating strong negative independent relationships between adiposity and PA, but with no evidence of any independent association between adiposity and EI. Despite some evidence in support of the EI methodology, its subjective nature and the paucity of quality studies limit our interpretation of the inability to detect any relationship with adiposity. In any case, this review-based evidence indicates that community-based strategies to prevent childhood and adolescent obesity in predominantly White, city-dwelling youth, would benefit by adopting a strong focus on increasing PA.

## Figures and Tables

**Table 1:** Methodological inclusion criteria.

Domain	Study Inclusion Criteria	Study Exclusion Criteria
Adiposity	Validated methods including dual-energy X-ray absorptiometry (DEXA), hydrostatic weighing, air displacement plethysmography (ADP), bioelectrical impedance analysis (BIA), and skinfold estimation of percent body fat.	Non-validated or indirect proxy measures of adiposity (e.g., BMI, waist circumference).
Energy Intake (EI)	Validated dietary assessment methods common to community-based studies, including 24 h dietary recall, food frequency questionnaire (FFQ), food diary/records, digital dietary assessment tools, and diet history interviews.	Absence of clear methodology, unvalidated self-reports.
Physical Activity (PA)	Validated methods included accelerometers, pedometers, heart rate monitors, and GPS devices.	Non-validated measures, e.g., unstructured self-report diaries and questionnaires.
Statistical treatment	Statistical regression models with adiposity as the response variable and PA and EI as explanatory variables, adjusting in turn for the covariates.	Simple correlations between adiposity and EI, and/or adiposity and PA.

**Table 2:** Attributes of the included studies.

Study	Participants	Measurements	Analyses	Some Key Findings
LOOK Pre-adolescence Study	534 (278 boys), mainly White, age 8 and 12 years, outer suburban mid-sized Australian city.	Pedometers, accelerometers; percent body fat (%BF) DEXA; 1-day dietary record at age 8 y, 2-day recall at 12 y).	General linear mixed method model, with adjustment for relevant covariates.	%BF independently and negatively associated with PA (*p* < 0.001); no association between %BF and EI (*p* = 0.3; no evidence of any effect of under-reporting in children with higher %BF.
Helena/European Youth Heart Study	1721 adolescents (792 boys): presumed mainly White from 10 cities in 9 European countries.	Accelerometry; skinfolds, ADP, DEXA, BIA; EI; 24 h dietary recall.	Multilevel analysis with adjustments for relevant covariates.	Fat mass was negatively associated with energy intake in both studies (≤0.006); More active adolescents were leaner with greater EI than less active adolescents, who had greater %BF; no evidence of any effect of under-reporting in children with higher %BF.
Iowa Bone Development Study	493 participants (243 boys), mainly White, were assessed at least 3 times from ages 5 to 19 years. USA.	PA: Accelerometry; adiposity: DEXA; EI: food frequency questionnaires.	Quartile ranking for EI; logistic regression models with adjustment for relevant covariates.	Declining PA was associated with increased odds of obesity (2.77; 95% CI, 1.16, 6.58); little or no association between EI and adiposity; with higher EI tending to lower obesity odds (95% CI 0.74, (0.39, 1.40)
LOOK Adolescence Study	556 participants (289 male) at age 12; 269 remeasured at age 16, from an outer suburban mid-sized Australian city.	Adiposity: DEXA; PA: accelerometry; EI: 2 × 24 h recall, weekday/weekend multi-pass methodology.	General linear mixed modeling with adjustments for relevant covariates.	%BF was independently negatively related to PA (*p* < 0.001); no relationship between %BF and energy intake (*p* = 0.4). Adjustments for any potential under-reporting in individuals with higher %BF; no evidence of under-reporting in children with higher %BF.

## Data Availability

Not applicable.

## Supplementary Materials

The following supporting information can be downloaded at: https://www.mdpi.com/article/10.3390/nu17233779/s1, Table S1. (a) Search concepts; (b) Exemplar search syntax; (c) Robins-E Risk of Bias Analyses.

## Abbreviations

The following abbreviations are used in this manuscript:

PA	Physical activity
EI	Energy intake
DEXA	Dual-energy X-ray absorptiometry
EYHS	European Youth Heart Study
BIA	Bio-electrical impedance analysis

## References

[1] Lister N.B., Baur L.A., Felix J.F., Hill A.J., Marcus C., Reinehr T., Summerbell C., Wabitsch M. (2023). Child and adolescent obesity. Nat. Rev. Dis. Primers.

[2] Qasim A., Turcotte M., de Souza R.J., Samaan M.C., Champredon D., Dushoff J., Speakman J.R., Meyre D. (2018). On the origin of obesity: Identifying the biological, environmental and cultural drivers of genetic risk among human populations. Obes. Rev..

[3] Lobstein T., Jackson-Leach R., Moodie M.L., Hall K.D., Gortmaker S.L., Swinburn B.A., James W.P.T., Wang Y., McPherson K. (2015). Child and adolescent obesity: Part of a bigger picture. Lancet.

[4] Simmonds M., Llewellyn A., Owen C.G., Woolacott N. (2016). Predicting adult obesity from childhood obesity: A systematic review and meta-analysis. Obes. Rev..

[5] Kerr J.A., Patton G.C., Cini K.I., Abate Y.H., Abbas N., Abd Al Magied A.H., Abd El Hafeez S., Abd-Elsalam S., Abdollahi A., Abdoun M. (2025). Global, regional, and national prevalence of child and adolescent overweight and obesity, 1990–2021, with forecasts to 2050: A forecasting study for the Global Burden of Disease Study 2021. Lancet.

[6] Walls H.L., Peeters A., Proietto J., McNeil J.J. (2011). Public health campaigns and obesity—A critique. BMC Public Health.

[7] Telford R.D., Cunningham R.B., Abhayaratna W. (2014). Temporal divergence of percent body fat and body mass index in pre-teenage children: The LOOK longitudinal study. Pediatr. Obes..

[8] Telford R.D., Telford R.M., Welvaert M. (2019). BMI is a misleading proxy for adiposity in longitudinal studies with adolescent males: The Australian LOOK study. J. Sci. Med. Sport.

[9] Telford R.D., Cunningham R.B., Telford R.M., Riley M., Abhayaratna W.P. (2012). Determinants of childhood adiposity: Evidence from the Australian LOOK study. PLoS ONE.

[10] Hands B., Parker H., Glasson C., Brinkman S., Read H. (2004). Results of Western Australian Child and Adolescent Physical Activity and Nutrition Survey 2003 (CAPANS. Physical Activity Technical Report).

[11] Cuenca-García M., Ortega F.B., Ruiz J.R., Labayen I., Moreno L.A., Patterson E., Vicente-Rodríguez G., González-Gross M., Marcos A., Polito A. (2014). More physically active and leaner adolescents have higher energy intake. J. Pediatr..

[12] Kwon S., Janz K.F., Letuchy E.M., Burns T.L., Levy S.M. (2015). Active lifestyle in childhood and adolescence prevents obesity development in young adulthood. Obesity.

[13] Goldberg G., Black A., Jebb S., Cole T., Murgatroyd P., Coward W., Prentice A. (1991). Critical evaluation of energy intake data using fundamental principles of energy physiology: 1. Derivation of cut-off limits to identify under-recording. Eur. J. Clin. Nutr..

[14] Joosen A.M., Westerterp K.R. (2006). Energy expenditure during overfeeding. Nutr. Metab..

[15] Byrne N.M., Hills A.P. (2013). Biology or behavior: Which is the strongest contributor to weight gain?. Curr. Obes. Rep..

[16] Bray G.A., Frühbeck G., Ryan D.H., Wilding J.P. (2016). Management of obesity. Lancet.

[17] Blundell J., Gibbons C., Caudwell P., Finlayson G., Hopkins M. (2015). Appetite control and energy balance: Impact of exercise. Obes. Rev..

[18] McCarthy S.F., Tucker J.A., Hazell T.J. (2024). Exercise-induced appetite suppression: An update on potential mechanisms. Physiol. Rep..

[19] Cade J.E., Warthon-Medina M., Albar S., Alwan N.A., Ness A., Roe M., Wark P.A., Greathead K., Burley V.J., Finglas P. (2017). DIET@NET: Best Practice Guidelines for dietary assessment in health research. BMC Med..

[20] Park Y., Dodd K.W., Kipnis V., Thompson F.E., Potischman N., Schoeller D.A., Baer D.J., Midthune D., Troiano R.P., Bowles H. (2018). Comparison of self-reported dietary intakes from the Automated Self-Administered 24-h recall, 4-d food records, and food-frequency questionnaires against recovery biomarkers. Am. J. Clin. Nutr..

[21] Hjorth M.F., Chaput J.-P., Ritz C., Dalskov S.-M., Andersen R., Astrup A., Tetens I., Michaelsen K.F., Sjödin A. (2014). Fatness predicts decreased physical activity and increased sedentary time, but not vice versa: Support from a longitudinal study in 8- to 11-year-old children. Int. J. Obes..

[22] Gutin B. (2011). Diet vs. exercise for the prevention of pediatric obesity: The role of exercise. Int. J. Obes..

