# Supplementary material for: Do Physical Activity and Diet Independently Account for Variation in Body Fat in Children and Adolescents? A Systematic Review Unpacking the Roles of Exercise and Diet in Childhood Obesity
Source: Nutrients. 2025 Dec 2;17(23):3779. doi: 10.3390/nu17233779 (PMC12693829; doi:10.3390/nu17233779)
Supplement: Supplementary file 1 [file nutrients-17-03779-s001.zip › nutrients-3989221-supplementary.pdf]

Supplementary Table S1a: Search concepts

	Concept 1	Concept 2	Concept 3	Concept 4	Concept 5
	**Physical activity> children> BC**	**Energy intake/expenditure>children BC**	**Objective BC**	**Objective energy intake and output**	**Objective physical activity**
Key words	Exercise; sedentary lifestyle; physical activity; fitness<br>Child*; adolescen*; youth; teen*<br>Body composition	Energy intake; energy expenditure; energy output; caloric intake; diet<br>Child*; adolescen*; youth; teen*<br>Body composition	Dual-energy X-ray absorptiometry<br>Air displacement plethysmography<br>Bioelectrical impedance analysis<br>Skinfold thickness<br>Hydrostatic weighing<br>Magnetic resonance<br>Computed tomography	Direct calorimetry<br>Indirect calorimetry<br>Doubly labeled water<br>Accelerometers<br>Heart rate monitors<br>Food diaries<br>24-hour dietary recall<br>Weighed food records	GPS<br>Pedometer<br>Doubly labeled water<br>Accelerometer*<br>Heart rate monitor*<br>Direct observation<br>Sensewear armband<br>Wearable*
Alternative terms	physical fitness;	carbohydrates; protein; fat; macronutrients; supplements; Energy Metabolism; metabolism;	anthropometry; body composition; body fat; body mass; obesity; weight gain; photon absorptiometry; Absorptiometry, Photon; Adiposity; Body Mass Index; obesity		

Supplementary Table S1b: Exemplar search syntax

Database	Search Query
Medline Ovid	((child* OR adolescen* OR youth OR teen*) AND (body AND composition) AND (exercise OR fitness OR sedentary OR physical activity)) OR ((child* OR adolescen* OR youth OR teen*) AND (body AND composition) AND ("energy intake" OR "caloric intake" OR "energy expenditure" OR diet)) AND (("Direct calorimetry" OR "Indirect calorimetry" OR "Doubly labeled water" OR Accelerometer* OR "Heart rate monitor*" OR "Food diaries" OR "24-hour dietary recall" OR "Weighed food records")) AND (gps OR pedometer OR "Doubly labeled water" OR Accelerometer* OR "Heart rate monitor*" OR "Direct observation" OR "Sensewear armband" OR Wearable*) AND (("Dual-energy X-ray absorptiometry" OR "Air displacement plethysmography" OR "Bioelectrical impedance" OR "Skinfold thickness" OR "Hydrostatic weighing" OR "Magnetic resonance" OR "Computed Tomography" OR "deuterium dilution"))
SPORTDiscus	((TI (child* OR adolescen* OR youth OR teen*) AND TI (body composition) AND (TI (exercise OR fitness OR sedentary OR physical activity))) OR (TI (child* OR adolescen* OR youth OR teen*) AND TI (body composition) AND TI ("energy intake" OR "caloric intake" OR "energy expenditure" OR diet))) AND (TI ("Direct calorimetry" OR "Indirect calorimetry" OR "Doubly labeled water" OR Accelerometer* OR "Heart rate monitor*" OR "Food diaries" OR "24-hour dietary recall" OR "Weighed food records")) AND (TI (gps OR pedometer OR "Doubly labeled water" OR Accelerometer* OR "Heart rate monitor*" OR "Direct observation" OR "Sensewear armband" OR Wearable*)) AND (TI ("Dual-energy X-ray absorptiometry" OR "Air displacement plethysmography" OR "Bioelectrical impedance" OR "Skinfold thickness" OR "Hydrostatic weighing" OR "Magnetic resonance" OR "Computed Tomography" OR "deuterium dilution"))
Web of Science	((TS=(child* OR adolescen* OR youth OR teen*) AND TS=(body AND composition) AND (TS=(exercise OR fitness OR sedentary OR "physical activity")) OR (TS=(child* OR adolescen* OR youth OR teen*) AND TS=(body AND composition) AND TS=("energy intake" OR "caloric intake" OR "energy expenditure" OR diet))) AND (TS=("Direct calorimetry" OR "Indirect calorimetry" OR "Doubly labeled water" OR Accelerometer* OR "Heart rate monitor*" OR "Food diaries" OR "24-hour dietary recall" OR "Weighed food records")) AND (TS=(gps OR pedometer OR "Doubly labeled water" OR Accelerometer* OR "Heart rate monitor*" OR "Direct observation" OR "Sensewear armband" OR Wearable*)) AND (TS=("Dual-energy X-ray absorptiometry" OR "Air displacement plethysmography" OR "Bioelectrical impedance" OR "Skinfold thickness" OR "Hydrostatic weighing" OR "Magnetic resonance" OR "Computed Tomography" OR "deuterium dilution"))
Scopus	((TITLE-ABS-KEY ((child* OR adolescen* OR youth OR teen*) AND (body AND composition) AND (exercise OR fitness OR sedentary OR (physical AND activity)))) OR (TITLE-ABS-KEY ((child* OR adolescen* OR youth OR teen*) AND (body AND composition) AND ("energy intake" OR "caloric intake" OR "energy expenditure" OR diet)))) AND (TITLE-ABS-KEY ("Direct calorimetry" OR "Indirect calorimetry" OR "Doubly labeled water" OR Accelerometer* OR "Heart rate monitor*" OR "Food diaries" OR "24-hour dietary recall" OR "Weighed food records")) AND (TITLE-ABS-KEY (gps OR pedometer OR "Doubly labeled water" OR Accelerometer* OR "Heart rate monitor*" OR "Direct observation" OR "Sensewear armband" OR Wearable*)) AND (TITLE-ABS-KEY ("Dual-energy X-ray absorptiometry" OR "Air displacement plethysmography" OR "Bioelectrical impedance" OR "Skinfold thickness" OR "Hydrostatic weighing" OR "Magnetic resonance" OR "Computed Tomography" OR "deuterium dilution"))

Supplementary Table S1c: Robins-E Risk of Bias Analyses

Domain	LOOK Pre-adolescence Study	Helena/European Youth Heart Study	Iowa Bone Development Study	LOOK Adolescence Study
Bias due to confounding	Moderate – PA, EI, maturation measured but residual lifestyle confounding likely	Moderate – Adjusted for age/puberty/center; residual confounding possible	Moderate–Serious – Long trajectories but limited control for socioeconomic/lifestyle factors	Moderate – Good PA/EI measurement but confounders only partially controlled
Bias in selection of participants into the study	Moderate – Only children completing measures at both ages included	Moderate – Large cohorts but volunteer/center selection variability	Moderate – Requirement for multi-wave participation may bias sample	Moderate – Attrition from age 12 to16 may bias sample
Bias in classification of exposures	Low–moderate – Pedometers and recalls; one vs. two days dietary recording differences	Moderate – PA by accelerometry; EI by 1–2 recalls; measurement variability	Moderate – Different EI questionnaires across waves	Low–Moderate – Accelerometry + validated 24-h recall with Goldberg cut-off
Bias due to missing data	Moderate – Excludes missing cases; limited detail on dropout	Moderate – Some missing PA/EI data; harmonisation challenges	Serious – Long follow-up with substantial attrition risk	Moderate – Only 269/556 followed to age 16
Bias in measurement of outcomes	Low – DEXA for adiposity	Low–Moderate – Mix of DEXA/BIA/skinfolds/BODPOD	Low – DEXA used consistently	Low – DEXA for %BF
Bias in selection of reported results	Moderate – Some outcomes not reported but main analyses prespecified	Moderate – Multiple PA/EI/adiposity metrics may allow selective reporting	Moderate – Trajectory choices may allow reporting flexibility	Moderate – Cross-sectional + longitudinal; selective model choice possible
Overall bias	Low-moderate	Low- moderate	Moderate	Low-moderate
